# Effect of cilostazol on plasma levels of proprotein convertase subtilisin/kexin type 9

**DOI:** 10.18632/oncotarget.22448

**Published:** 2017-11-14

**Authors:** I-Chih Chen, Wei-Kung Tseng, Yi-Heng Li, Shih-Ya Tseng, Ping-Yen Liu, Ting-Hsing Chao

**Affiliations:** ^1^ Department of Internal Medicine, Tainan Municipal Hospital, Tainan, Taiwan; ^2^ Department of Medical Imaging and Radiological Sciences, I-Shou University and Division of Cardiology, Department of Internal Medicine, E-Da Hospital, Kaohsiung, Taiwan; ^3^ Department of Internal Medicine, National Cheng Kung University College of Medicine and Hospital, Tainan, Taiwan; ^4^ Institute of Clinical Medicine, College of Medicine, National Cheng Kung University, Tainan, Taiwan

**Keywords:** cilostazol, proprotein convertase subtilisin/kexin type 9, lipids, peripheral artery disease

## Abstract

The protein complex proprotein convertase subtilisin/kexin type 9 (PCSK9) serves as an important target for the prevention and treatment of atherosclerosis and lipid homeostasis. This study investigated the effect of cilostazol on plasma PCSK9 concentrations. We performed a post hoc analysis of two prospective, double-blind, randomized controlled trials including 115 patients of whom 61 received cilostazol 200 mg/day and 54 received placebo for 12 weeks. Linear regression analysis was performed to determine the associations between several parameters and changes in PCSK9 levels. Use of cilostazol, but not placebo, significantly increased plasma PCSK9 concentrations, high-density lipoprotein cholesterol levels, and number of circulating endothelial progenitor cells (EPCs), and decreased triglyceride levels with a trend toward an increase in total cholesterol (TC) levels. A reduction in hemoglobin A1C and an increase in plasma vascular endothelial growth factor and adiponectin levels with cilostazol treatment were also found. Changes in the number of circulating EPCs were positively correlated and the TC concentrations were inversely correlated with changes in the PCSK9 levels. After adjusting for changes in levels of TC and numbers of circulating EPCs and history of metabolic syndrome, use of cilostazol remained independently associated with changes in plasma PCSK9 levels. In conclusion, cilostazol treatment was significantly and independently associated with an increase in plasma PCSK9 levels in patients with peripheral artery disease or at a high risk of cardiovascular disease regardless of background statin use and caused an improvement in some metabolic disorders and levels of vasculo-angiogenic biomarkers.

## INTRODUCTION

Proprotein convertase subtilisin/kexin type 9 (PCSK9), a newly recognized protein, plays an important role in cholesterol homeostasis and affects plasma lipoprotein levels by enhancing the degradation of hepatic low-density lipoprotein receptors and subsequently reducing expression of these receptors on hepatocytes, thereby leading to increased plasma levels of total cholesterol (TC) and low-density lipoprotein cholesterol (LDL-C) [1]. From a genetic point of view, individuals with certain sequence variations in the *PCSK9* gene have not only lower plasma LCL-C levels but also a lower incidence of coronary artery disease (CAD) [2]. Plasma PCSK9 levels are not only correlated with atherogenic lipoprotein levels but also with many other cardiovascular risk factors, such as fasting plasma glucose, age, and blood pressure [3, 4]. In addition to traditional risk factors, PCSK9 is also associated with non-traditional risk factors involving inflammatory and oxidative processes [5–9]. Moreover, plasma PCSK9 levels have been reported to be associated with the severity of CAD [10], and the presence of peripheral artery disease (PAD), especially those with extensive, severe, and complicated PAD [11]. We found that circulating endothelial progenitor cell (EPC) dysfunction, in particular, the number of apoptotic circulating endothelial cells, and some vasculo-angiogenic and oxidative biomarkers were significantly correlated with PCSK9 levels [11]. Taken together, PCSK9 protein is an important target for the prevention and treatment of atherosclerosis.

Some studies have revealed that plasma PCSK9 levels were affected by some drugs, such as an increase in PCSK9 levels with the use of statins [12], ezetimibe [13], and fibrates [14], and a decrease with nicotinic acid treatment [15]. Recently, some studies have indicated a relationship between PCSK9 and the metabolism of high-density lipoprotein cholesterol (HDL-C) [16] and triglyceride [17].

Cilostazol, a phosphodiesterase 3 inhibitor, is licensed for treatment of patients with PAD and intermittent claudication owing to its antiplatelet and vasodilatory effects [18–20]. Recently, we and other researchers found that this compound has beneficial effects on metabolic parameters, angiogenesis, numbers and functions of circulating human early EPCs *in vitro* [18, 21–24], *in vivo* [18, 21–24], and in clinical settings [19, 20]. In particular, cilostazol treatment is beneficial for a reduction in triglyceride levels and an increase in HDL-C levels in patients with PAD [19, 25] or at high risk of cardiovascular disease (CVD) [20]. However, no study has investigated the effect of cilostazol on plasma PCSK9 levels.

In our study, we included participants from two double-blind, randomized, placebo-controlled trials [19, 20] to evaluate the effect of cilostazol treatment on plasma PCSK9 levels and the potential mechanisms of action in patients with PAD or at high risk of CVD.

## RESULTS

**Figure 1:**
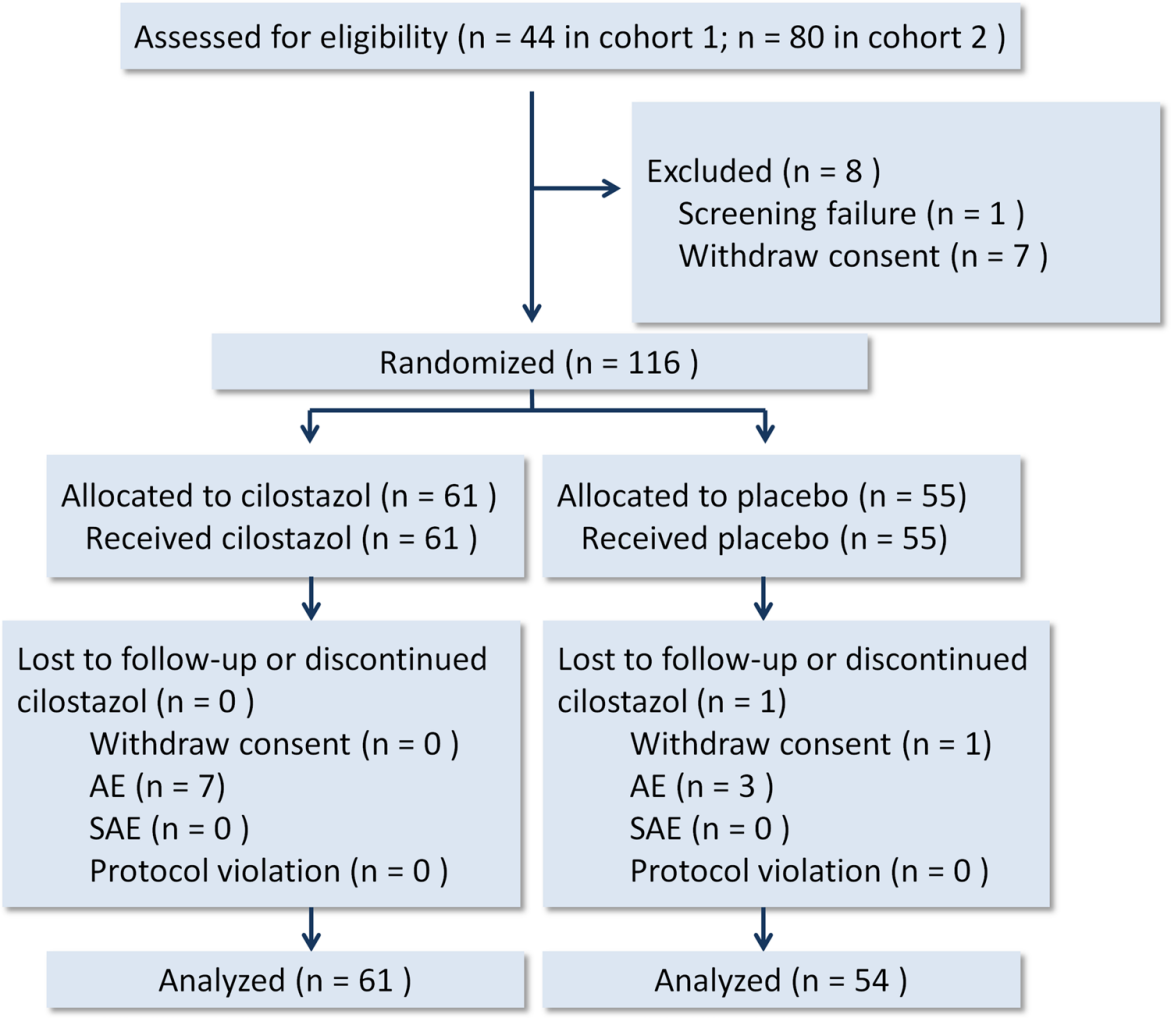
Participant flow diagram AE, adverse event. SAE, serious adverse event.

The participant flow of the two cohorts is demonstrated in Figure 1. The mean patient age was 65.6 ± 9.3 years, and 66.1% of the patients were male. The background characteristics and parameters between the cilostazol treatment and placebo groups were similar and well matched (Table 1). All participants could tolerate the treatment protocol and completed the entire study.

**Table 1:** Baseline characteristics and parameters between patients in the cilostazol treatment and placebo groups

Variables	Cilostazol group (*n* = 61)	Placebo group (*n* = 54)	*P* value
Age, years	65.5 ± 1.3	65.6 ± 1.2	0.95
Male gender	38 (62.3)	38 (70.4)	0.36
Underlying disease			
PAD	24 (39.3)	20 (37.0)	0.80
CAD	16 (26.2)	14 (25.9)	0.97
Old myocardial infarction	10 (16.4)	3 (5.6)	0.07
Old cerebrovascular accident	5 (8.2)	3 (5.6)	0.72
Diabetes mellitus	30 (49.2)	23 (42.6)	0.48
Hypertension	48 (78.7)	40 (74.1)	0.56
Hyperlipidemia	47 (77.0)	39 (72.2)	0.55
Metabolic syndrome	37 (60.7)	33 (61.1)	0.96
Tobacco smoking	10 (16.4)	13 (24.1)	0.30
Peptic ulcer disease	2 (3.3)	3 (5.6)	0.66
Chronic kidney disease	6 (9.8)	7 (13.0)	0.60
Aspirin use	37 (60.7)	23 (42.6)	0.06
Clopidogrel use	3 (4.9)	5 (9.3)	0.47
ACEI use	10 (16.4)	10 (18.5)	0.76
ARB use	29 (47.5)	22 (40.7)	0.46
CCB use	37 (60.7)	25 (46.3)	0.12
Statin use	33 (54.1)	23 (42.6)	0.22
Thiazolidinedione use	5 (8.2)	7 (13.0)	0.40
Fasting plasma glucose, mg/dL	126.3 ± 8.2	112.5 ± 3.9	0.13
Hemoglobin A1C, %	6.7 ± 0.2	6.5 ± 0.1	0.32
Fasting insulin, mU/L	9.7 (6.7, 15.6)	9.7 (5.5, 17.7)	0.86
HOMA index	2.6 (1.5, 5.3)	2.4 (1.4, 5.0)	0.80
Body weight, kg	72.0 ± 1.5	73.0 ± 2.1	0.68
Waist circumference, cm	97.0 ± 1.2	96.9 ± 1.6	0.93
Body mass index, kg/m^2^	28.1 ± 0.5	27.8 ± 0.7	0.74
Blood pressure, mmHg			
Systolic	136.6 ± 2.4	130.9 ± 2.1	0.07
Diastolic	80.5 ± 2.0	76.5 ± 1.5	0.11
Heart rate, beats/min	78.1 ± 1.7	77.4 ± 1.9	0.79
White blood cell count, 10^3^/μL	6550.8 ± 213.3	6557.4 ± 266.3	0.98
Hemoglobin, g/dL	13.8 ± 0.2	14.1 ± 0.5	0.57
Platelet count, 10^3^/μL	201.2 ± 5.4	206.5 ± 6.4	0.52
TC, mg/dL	177.0 (152.0, 194.5)	185.5 (163.8, 205.5)	0.12
Triglyceride, mg/dL	144.8 ± 14.4	151.4 ± 13.1	0.74
HDL-C, mg/dL	53.1 ± 1.7	49.4 ± 1.6	0.13
LDL-C, mg/dL	110.3 ± 4.0	119.0 ± 4.7	0.16
Colony-forming units, /1×10^6^ PBMCs	48.9 ± 5.0	53.7 ± 6.6	0.56
KDR^+^CD34^+^ count, cells/μL	0.42 (0.10, 1.62)	0.24 (0.11, 1.48)	0.65
CD146^+^annexin V^+^ count, cells/μL	0.03 (0.02, 0.09)	0.04 (0.02, 0.06)	0.91
BrDU incorporation, absorbance value at 450 nm	1.0 ± 0.1	1.0 ± 0.1	0.84
XTT, absorbance value at 450 nm	2.2 (1.7, 2.7)	2.2 (1.8, 2.6)	0.79
Nucleosome fragmentation, fold	1.6 ± 0.1	1.5 ± 0.1	0.23
Migrated cells, /field	155.2 ± 14.3	134.9 ± 14.2	0.32
Soluble TM, pg/mL	5045.9 ± 308.9	6490.9 ± 992.4	0.17
VEGF-A165, pg/mL	351.0 ± 34.3	442.9 ± 50.3	0.14
SDF-1ɑ, pg/mL	1927.7 ± 142.7	2143.9 ± 160.6	0.32
Adiponectin, ng/mL	5099.5 ± 540.1	5403.8 ± 576.7	0.70
PCSK9, ng/mL	375.2 ± 23.6	358.0 ± 24.2	0.61

ACEI, angiotensin-converting enzyme inhibitor. ARB, angiotensin receptor blocker. CCB, calcium channel blocker. HOMA, homeostasis model assessment. PBMC, peripheral blood mononuclear cell. SDF-1ɑ, stromal cell-derived factor-1ɑ. TM, thrombomodulin.

Numerical values are expressed as mean ± standard deviation or median (interquartile range), as appropriate, and categorical variables are expressed as number (%).

**Figure 2:**
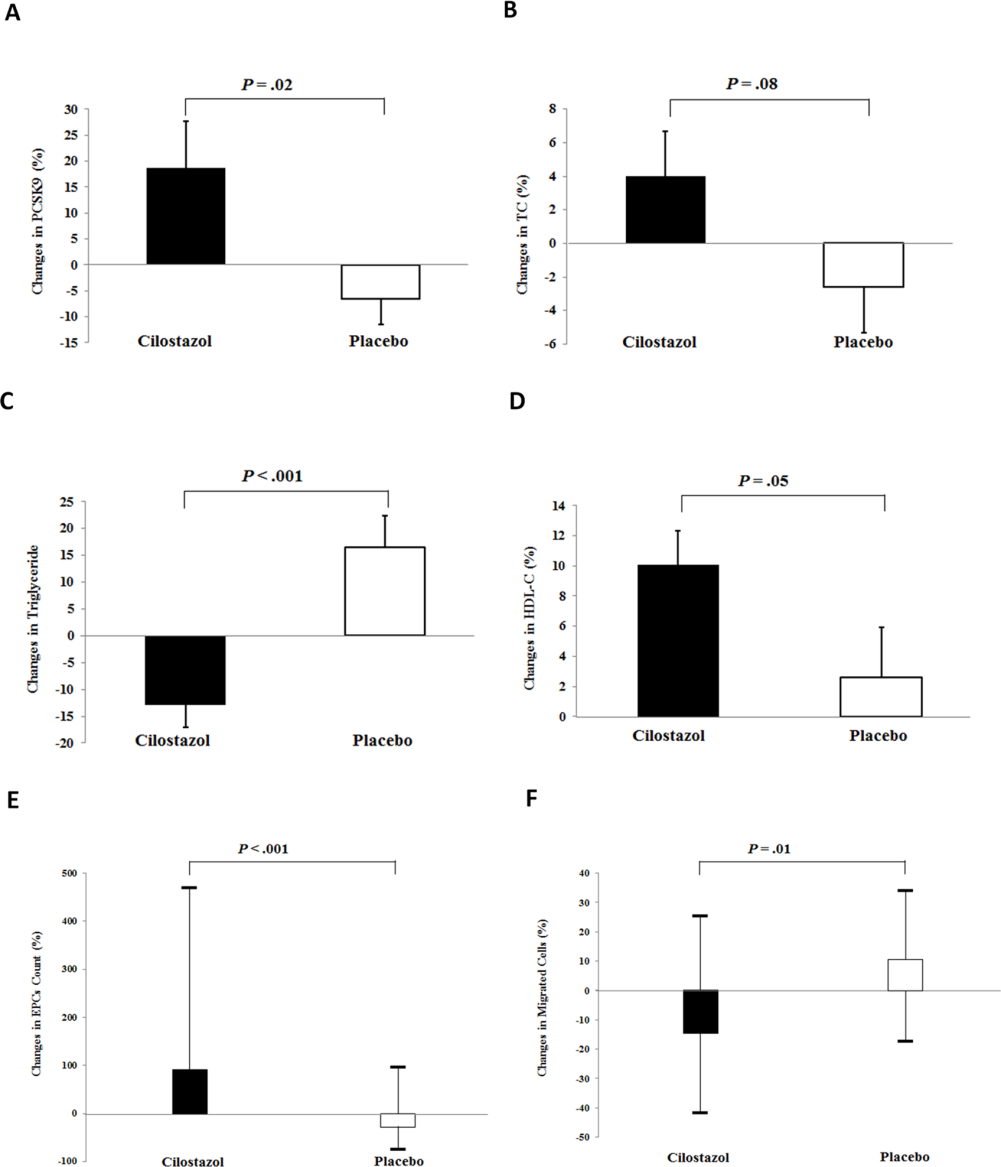
Effects of cilostazol on proprotein convertase subtilisin/kexin type 9 (PCSK9) concentrations, lipid parameters, and circulating number and functions of endothelial progenitor cells (EPCs) Our data revealed the effects of cilostazol treatment in **(A)** PCSK9 concentrations, **(B)** total cholesterol (TC) levels, **(C)** triglyceride levels, **(D)** high-density lipoprotein cholesterol (HDL-C) levels, **(E)** circulating number of EPCs, and **(F)** migrated EPCs. Values are expressed as mean ± standard deviation or median (interquartile range), as appropriate.

Comparisons of changes in the background parameters, number of circulating EPCs and apoptotic endothelial cells, *in vitro* EPC functions, and serum/plasma biomarker levels, after treatment between the groups are demonstrated in Table 2 and Figure 2. Cilostazol treatment significantly increased plasma PCSK9 levels (18.7 ± 8.9% vs. -6.5 ± 5.1%, *P* = 0.02) and improved triglyceride and HDL-C levels (-13.0 ± 4.0% vs. 16.5 ± 5.8%, *P* < 0.001; 10.1 ± 2.2% vs. 2.6 ± 3.3%, *P* = 0.05, respectively). Despite no statistically significant changes in the TC and LDL-C levels between the groups, cilostazol treatment led to a marginal increase in TC levels. Cilostazol had a beneficial effect on glucose homeostasis, as shown by a significant improvement in hemoglobin A1C levels and a trend toward a decrease in fasting insulin levels and homeostasis model assessment index. Use of cilostazol, but not placebo, significantly increased the circulating EPC count [91.2 (25.9, 469.2)% vs. -28.1 (-76.0, 95.0)%, *P* < 0.001) and reduced migration capacity of EPCs, without any effects on the apoptotic endothelial cell count or other EPC functions. Cilostazol treatment tended to enhance EPC 2,3-bis-(2-methoxy-4-nitro-5-sulfophenyl)-2H-tetrazolium-5-carboxanilide (XTT) absorbance, a marker of cell viability (*P* = 0.06). Vascular endothelial growth factor (VEGF)-A165 and adiponectin concentrations significantly increased in the cilostazol treatment group [41.8 (16.1, 91.0)% vs. -6.6 (-39.9, 15.5)%, *P* < 0.001; 10.5 (-21.1, 50.9)% vs. -18.8 (-35.5, 5.0)%, *P* = 0.002, respectively], whereas other serum/plasma biomarker levels did not change significantly. Moreover, heart rate and platelet counts significantly increased with cilostazol treatment.

**Table 2:** Changes in parameters between patients in the cilostazol treatment and placebo groups

Changes in parameters^a^	Cilostazol group (*n* = 61)	Placebo group (*n* = 54)	*P* value
Fasting plasma glucose, %	1.1 ± 2.8	5.4 ± 2.9	0.29
Hemoglobin A1C, %	-1.8 ± 1.3	2.6 ± 1.4	0.03
Fasting insulin, %	7.4 ± 7.1	36.4 ± 15.0	0.09
HOMA index, %	12.0 ± 8.2	50.0 ± 19.6	0.08
Body weight, %	0.4 ± 3.3	0.7 ± 2.1	0.94
Waist circumference, %	-2.0 ± 0.6^c^	-2.1 ± 0.7^c^	0.95
Body mass index, %	28.5 ± 29.2	14.9 ± 13.4	0.68
Blood pressure, %			
Systolic	-0.9 ± 1.6	0.8 ± 1.2	0.41
Diastolic	14.5 ± 14.5	0.6 ± 1.6	0.35
Heart rate, %	9.9 ± 2.0^c^	-0.3 ± 1.9	< 0.001
White blood cell count, %	-3.2 ± 3.0	7.2 ± 6.5	0.14
Hemoglobin, %	-1.3 ± 0.8	-3.4 ± 2.1	0.35
Platelet count, %	5.3 ± 1.6^c^	0.1 ± 1.4	0.02
TC, %	4.0 ± 2.7	-2.6 ± 2.7	0.08
Triglyceride, %	-13.0 ± 4.0^c^	16.5 ± 5.8	< 0.001
HDL-C, %	10.1 ± 2.2^c^	2.6 ± 3.3	0.05
LDL-C, %	5.4 ± 4.8	0.3 ± 3.1	0.37
Colony-forming units, %	60.2 ± 25.9	93.3 ± 27.9	0.39
KDR^+^CD34^+^ cells, %	91.2 (25.9, 469.2)^c^	-28.1 (-76.0, 95.0)	< 0.001
CD146^+^annexin V^+^ cells, %	-33.3 (-76.0, 75.0)	0 (-48.1, 100.0)	0.13
BrDU incorporation, %	26.3 ± 10.6	15.1 ± 10.7	0.46
XTT absorbance, %	32.6 ± 14.3	3.5 ± 5.7	0.06
Nucleosome fragmentation, %	5.5 (-41.8, 49.9)	6.1 (-26.0, 83.4)	0.47
Migrated cells, %	-14.7 (-41.8, 25.3)^b^	10.6 (-17.5, 34.0)	0.01
Soluble TM, %	4.6 ± 4.5	14.9 ± 8.3	0.27
VEGF-A165, %	41.8 (16.1, 91.0)^c^	-6.6 (-39.9, 15.5)^b^	< 0.001
SDF-1ɑ, %	18.1 (-28.9, 65.6)	3.8 (-24.0, 57.5)	0.58
Adiponectin, %	10.5 (-21.1, 50.9)	-18.8 (-35.5, 5.0)^c^	0.002
PCSK9, %	18.7 ± 8.9^b^	-6.5 ± 5.1^b^	0.02

Abbreviations as shown in Table 1.

^a^Changes in parameters = (post-treatment value – baseline value) / baseline value × 100%. Values are expressed as mean ± standard deviation, number (%), or median (interquartile range), as appropriate.

^b^*P* < 0.05 vs. baseline. ^c^*P* < 0.01 vs. baseline.

Table 3 demonstrates that changes in PCSK9 plasma levels were similar with respect to the background characteristics or medications, except for history of metabolic syndrome. Patients with a history of metabolic syndrome had smaller changes in PCSK9 concentrations than other patients. Of note, changes in PCSK9 levels was similar in patients with or without statin treatment during the 3-month study period (3.9 ± 7.2% vs. 9.7 ± 8.1%, *P* = 0.60).

**Table 3:** The association of baseline characteristics and changes in plasma levels of PCSK9 in the entire cohort

	Changes in PCSK9 levels^a^ (%)		*P* value
	In patients with history of	In patients without history of	
Male gender	8.5 ± 7.2	3.7 ± 7.7	0.68
Underlying disease			
Diabetes mellitus	0.6 ± 7.1	12.3 ± 8.0	0.29
Hypertension	7.0 ± 6.4	6.3 ± 10.1	0.96
Hyperlipidemia	9.2 ± 6.5	-0.1 ± 9.5	0.46
Metabolic syndrome	-1.7 ± 6.1	20.2 ± 9.9	0.05
Tobacco smoking	-2.3 ± 11.9	9.2 ± 6.1	0.40
PAD	-2.2 ± 8.0	12.5 ± 7.2	0.19
CAD	-4.8 ± 9.6	11.0 ± 6.5	0.20
Myocardial infarction	4.1 ± 16.6	7.2 ± 5.8	0.86
Cerebrovascular accident	19.4 ± 29.9	5.9 ± 5.4	0.53
Chronic kidney disease	-5.2 ± 11.9	8.4 ± 5.9	0.43
Aspirin use	9.8 ± 7.1	3.7 ± 8.3	0.58
Clopidogrel use	-0.9 ± 14.3	7.4 ± 5.7	0.70
RASI use	12.5 ± 7.4	-1.9 ± 7.8	0.20
CCB use	1.7 ± 6.0	12.9 ± 9.5	0.32
Diuretic use	-6.1 ± 7.2	11.0 ± 6.7	0.18
Statin use	3.9 ± 7.2	9.7 ± 8.1	0.60

Abbreviations as shown in Table 1. RASI, renin-angiotensin system inhibitor.

^a^Changes in parameters = (post-treatment value – baseline value) / baseline value × 100%. Values are expressed as mean ± standard deviation.

Changes in the number of circulating EPCs was positively correlated, and the TC concentration was inversely correlated, with changes in PCSK9 levels (*r* = 0.21, *P* = 0.04; *r* = -0.22, *P* = 0.03, respectively) (Table 4). After adjusting for changes in TC levels, numbers of circulating EPCs, and history of metabolic syndrome, use of cilostazol remained independently associated with changes in the plasma PCSK9 levels [*beta* = 0.20 (95% confidence interval = 0.02-0.48), *P* = 0.04] (Table 5).

**Table 4:** Correlation between changes in PCSK9 levels and significant changes of parameters in the entire cohort

	Changes in PCSK9 levels	
	r	*P* value
Changes in TC levels	-0.22	0.03
Changes in KDR^+^CD34^+^ cells	0.21	0.04

Abbreviations as shown in Table 1.

**Table 5:** Independent predictors of changes in plasma levels of PCSK9 by multivariable linear regression analysis in the entire cohort

	Changes in PCSK9 levels	
	*Beta* (95% CI)	*P* value
Cilostazol use	0.20 (0.02, 0.48)	0.04
History of metabolic syndrome	-0.19 (-0.47, -0.01)	0.04
Changes in TC levels	-0.27 (-1.43, -0.26)	0.01
Changes in KDR^+^CD34^+^ cells	0.19 (0, 0.02)	0.05

Abbreviations as shown in Table 1.

## DISCUSSION

By evaluating two prospective, randomized, double-blinded, placebo-controlled trials in the current post hoc analysis, we found, for the first time, that cilostazol treatment was significantly and independently associated with an increase in plasma PCSK9 levels in patients with PAD or at a high risk of CVD regardless of background statin use. Although cilostazol treatment exerted a beneficial effect on some metabolic disorders and vasculo-angiogenic biomarkers, the increase in PCSK9 concentrations with this drug were probably mediated through other mechanisms. To the best of our knowledge, these findings have never been reported.

To date, no clinical trial has investigated the effects of cilostazol on plasma PCSK9 levels. Statins [12] and fibrates [14] significantly increase PCSK9 concentrations via upregulation of sterol regulatory element-binding protein-2 (SREBP-2). Fibrates may also function via an adiponectin-inducing effect [14]. In contrast, the mechanisms for ezetimibe and niacin are not well known [13, 15]. The ability of niacin to decrease PCSK9 concentrations might be attributed to a reduction in triglyceride synthesis and very low-density lipoprotein particle secretion from the liver [26]. The mechanism underlying the effect of cilostazol on PCSK9 concentrations is poorly understood and could only be speculated. Interestingly, the expression of PCSK9 can be regulated not only by SREBP-2 but also by peroxisome proliferator-activated receptor-γ (PPARγ) [27, 28]. Furthermore, activation of the adiponectin receptor can upregulate PCSK9 expression through activation of PPARγ and the adenosine monophosphate-activated protein kinase signaling pathway [28]. Our previous study revealed that cilostazol could serve as an activator of adenosine monophosphate-activated protein kinase signaling molecules [21] and adiponectin receptors (unpublished data), and our current study demonstrates that cilostazol treatment can increase plasma adiponectin concentrations. In addition, Sanada et al. found that induction of angiogenesis by cilostazol was through activation of PPARγ [29].

According to the current study, a history of metabolic syndrome and changes in TC levels and EPCs count are independently associated with changes in plasma PCSK9 concentrations. The mechanism responsible for this finding is not well known and probably very complex. It might be due to mixed effects of concomitant medications and disease, and inverse cause-and-effect relationships. A reduction in TC can lead to an increase in circulating EPC count [30] and might be, on the other hand, associated with increased PCSK9 concentrations due to less PCSK9 use up in certain situations [31]. Furthermore, PCSK9 could probably increase the circulating EPC count via the activation of PPARγ [28] and thereafter VEGF [11, 32], an enhancer of EPC mobilization from bone marrow [33].

The effect of cilostazol on TC and LDL-C is controversial. In the current study, cilostazol treatment had marginally increased TC levels, possibly due to the stimulating effect of cilostazol on PCSK9. Currently, the prognostic role of PCSK9 concentrations in cardiovascular outcomes remains a subject of debate [34–36]. Nevertheless, in light of the fact that PCSK9 is an important target for the prevention and treatment of atherosclerosis and lipid homeostasis [6], the clinical effect of additional PCSK9 inhibition during cilostazol treatment warrants further investigation.

This study was limited by the small sample size and short length of treatment, leading to a decrease in statistical power. Owing to the nature of post hoc analyses, we could not exclude the confounding effects of some relevant factors despite adjusting for multiple variables.

In considering the effects of cilostazol treatment on some metabolic disorders, vasculo-angiogenic biomarkers, and PCSK9 concentrations, cilostazol use might have a prognostic impact in patients with PAD or at a high risk of CVD. Further large-scale studies examining the effect of cilostazol use on cardiovascular outcomes and the clinical effect of additional PCSK9 inhibition during cilostazol treatment warrants further investigation.

## MATERIALS AND METHODS

### Patient population

This post hoc analysis was performed using two previous prospective, double-blind, randomized, placebo-controlled trials, which consecutively enrolled eligible patients with PAD (*n* = 44; started in January 2012 and completed in September 2013; cohort 1: ClinicalTrials.gov identifier: NCT01952756) or at high risk of CVD without pre-existing atherosclerotic disease (*n* = 71; started in January 2013 and completed in August 2014; cohort 2: ClinicalTrials.gov identifier: NCT02194686). The results of both studies have been published [19, 20]. The detailed inclusion and exclusion criteria were described previously [19, 20]. Briefly, patients who had mild-to-moderate PAD identified by ankle-brachial indices (< 0.9) but without obvious symptoms of intermittent claudication or critical limb ischemia were enrolled in cohort 1, whereas patients who had multiple cardiovascular risk factors but not pre-existing atherosclerotic diseases such as PAD or CAD were included in cohort 2. The current study without necessity of obtaining informed consent was conducted according to the Declaration of Helsinki and under the approval of the Ethics Committee of National Cheng Kung University Hospital (IRB number: A-ER-104-345).

### Randomization and allocation to treatment

During a 1-week run-in period, 124 eligible patients were screened (44 in cohort 1 and 80 in cohort 2). Eight patients were excluded prior to randomization (7 consent withdrawal; 1 screening failure due to active malignancy). During the 12-week study period, the eligible patients were randomly assigned to receive either 200 mg cilostazol (*n* = 61) or placebo (*n* = 55) daily (Figure 1) via the unrestricted randomization method with sealed envelopes for allocation concealment. The patients, care providers, and those who administered the interventions or performed assays were all blinded after assignment to interventions. One patient in the placebo group withdrew informed consent after treatment.

### Measurement of serum or plasma biomarkers

The baseline blood samples were obtained from the peripheral veins in all participants before treatment and the final samples were obtained using the same procedure one day immediately after the end of the study period. The blood samples were sent for isolation, cell culture, and assay of human EPCs, and prepared and stored for use in an enzyme-linked immunosorbent assay, as described previously [11, 19, 20, 37, 38]. Plasma or serum concentrations of biomarkers were measured using commercial kits (R&D Systems Inc., Minneapolis, MN, USA; and Mercodia AB, Uppsala, Sweden).

### Isolation and culture of EPCs, and determination of numbers of circulating EPCs and apoptotic endothelial cells

Isolation of early EPCs was performed by Ficoll density gradient centrifugation according to standard protocols, and colony formation by EPCs was identified and quantified, as described previously [11, 19–21]. EPCs were defined as CD45^-^CD34^+^ kinase insert domain receptor (KDR)^+^ cells, and circulating endothelial cells were defined as CD45^-^CD146^+^ annexin V^+^ cells [11, 20]. All fluorescence-labeled antibodies were purchased from Becton Dickinson (Arlington, VA, USA).

### Measurement of EPC functions *in vitro*

As previously described [11, 19–21], EPC migration was measured using modified Boyden chambers, proliferation and viability were assessed by bromodeoxyuridine (BrdU) and XTT assays, and EPC apoptosis was determined using a terminal 2′-deoxyuridine 5′-triphosphate nick end labeling assay kit (Roche, Basel, Switzerland).

### Primary measurement

As reported previously, the primary endpoint of both studies was the number of circulating EPCs, and the secondary endpoint was the viability of EPCs [19, 20]. The sample size estimation and power calculation were performed accordingly and described previously [20]. However, the current post hoc analysis aimed to detect the effect of cilostazol treatment on plasma PCSK9 levels as the primary measurement.

### Statistical analysis

Each analysis was performed in a per-protocol manner. Distributions of numerical variables in both groups were expressed as mean ± standard deviation, and skewed data were reported as median (interquartile range). Chi-square or Fisher's exact test were performed for comparing categorical variables between groups, whereas the Mann-Whitney *U* test or unpaired Student's *t*-test were performed for comparing numerical variables, as appropriate. Differences between baseline and post-treatment values were analyzed by the Wilcoxon signed- rank test or paired Student's *t*-test, as appropriate. The Pearson correlation was used to assess the relationship between the changes in metabolic factor levels in the plasma or serum, vasculoangiogenic biomarkers, EPC parameters, and changes in plasma PCSK9 levels after treatment over the entire cohort. The independent correlation of cilostazol use with the changes in plasma PCSK9 levels was adjusted for all uni-variables (a *P* value < 0.1) with a multivariable linear regression model by a stepwise regression method. A *P* < 0.05 (2-sided) was considered to indicate statistical significance. All statistical analyses were performed using SPSS for Windows (Version 13.0, SPSS Inc., Chicago, IL, USA).

## References

[1] Li S, Li JJ (2015). PCSK9: a key factor modulating atherosclerosis. J Atheroscler Thromb.

[2] Cohen JC, Boerwinkle E, Mosley TH, Hobbs HH (2006). Sequence variations in PCSK9, low LDL, and protection against coronary heart disease. N Engl J Med.

[3] Baass A, Dubuc G, Tremblay M, Delvin EE, O’Loughlin J, Levy E, Davignon J, Lambert M (2009). Plasma PCSK9 is associated with age, sex, and multiple metabolic markers in a population-based sample of children and adolescents. Clin Chem.

[4] Cui Q, Ju X, Yang T, Zhang M, Tang W, Chen Q, Hu Y, Haas JV, Troutt JS, Pickard RT, Darling R, Konrad RJ, Zhou H (2010). Serum PCSK9 is associated with multiple metabolic factors in a large Han Chinese population. Atherosclerosis.

[5] Feingold KR, Moser AH, Shigenaga JK, Patzek SM, Grunfeld C (2008). Inflammation stimulates the expression of PCSK9. Biochem Biophys Res Commun.

[6] Urban D, Pöss J, Böhm M, Laufs U (2013). Targeting the proprotein convertase subtilisin/kexin type 9 for the treatment of dyslipidemia and atherosclerosis. J Am Coll Cardiol.

[7] Zhang Y, Zhu CG, Xu RX, Li S, Guo YL, Sun J, Li JJ (2014). Relation of circulating PCSK9 concentration to fibrinogen in patients with stable coronary artery disease. J Clin Lipidol.

[8] Li S, Guo YL, Xu RX, Zhang Y, Zhu CG, Sun J, Qing P, Wu NQ, Jiang LX, Li JJ (2014). Association of plasma PCSK9 levels with white blood cell count and its subsets in patients with stable coronary artery disease. Atherosclerosis.

[9] Ding Z, Liu S, Wang X, Deng X, Fan Y, Shahanawaz J, Shmookler Reis RJ, Varughese KI, Sawamura T, Mehta JL (2015). Cross-talk between LOX-1 and PCSK9 in vascular tissues. Cardiovasc Res.

[10] Li S, Guo YL, Xu RX, Zhang Y, Zhu CG, Sun J, Qing P, Wu NQ, Li JJ (2014). Plasma PCSK9 levels are associated with the severity of coronary stenosis in patients with atherosclerosis. Int J Cardiol.

[11] Chao TH, Chen IC, Li YH, Lee PT, Tseng SY (2016). Plasma levels of proprotein convertase subtilisin/kexin type 9 are elevated in patients with peripheral artery disease and associated with metabolic disorders and dysfunction in circulating progenitor cells. J Am Heart Assoc.

[12] Sahebkar A, Simental-Mendía LE, Guerrero-Romero F, Golledge J, Watts GF (2015). Effect of statin therapy on plasma proprotein convertase subtilisin kexin 9 (PCSK9) concentrations: a systematic review and meta-analysis of clinical trials. Diabetes Obes Metab.

[13] Davignon J, Dubuc G (2009). Statins and ezetimibe modulate plasma proprotein convertase subtilisin kexin-9 (PCSK9) levels. Trans Am Clin Climatol Assoc.

[14] Sahebkar A (2014). Circulating levels of proprotein convertase subtilisin kexin type 9 are elevated by fibrate therapy: a systematic review and meta-analysis of clinical trials. Cardiol Rev.

[15] Croyal M, Ouguerram K, Passard M, Ferchaud-Roucher V, Chétiveaux M, Billon-Crossouard S, de Gouville AC, Lambert G, Krempf M, Nobécourt E (2015). Effects of extended-release nicotinic acid on apolipoprotein (a) kinetics in hypertriglyceridemic patients. Arterioscler Thromb Vasc Biol.

[16] Choi S, Korstanje R (2013). Proprotein convertases in high-density lipoprotein metabolism. Biomark Res.

[17] Druce I, Abujrad H, Ooi TC (2015). PCSK9 and triglyceride-rich lipoprotein metabolism. J Biomed Res.

[18] Chao TH, Tseng SY, Li YH, Liu PY, Cho CL, Shi GY, Wu HL, Chen JH (2012). A novel vasculo-angiogenic effect of cilostazol mediated by cross-talk between multiple signalling pathways including the ERK/p38 MAPK signaling transduction cascade. Clin Sci (Lond).

[19] Chao TH, Tseng SY, Chen IC, Tsai YS, Huang YY, Liu PY, Ou HY, Li YH, Wu HL, Cho CL, Tsai LM, Chen JH (2014). Cilostazol enhances mobilization and proliferation of endothelial progenitor cells and collateral formation by modifying vasculo-angiogenic biomarkers in peripheral arterial disease. Int J Cardiol.

[20] Chao TH, Chen IC, Lee CH, Chen JY, Tsai WC, Li YH, Tseng SY, Tsai LM, Tseng WK (2016). Cilostazol enhances mobilization of circulating endothelial progenitor cells and improves endothelium-dependent function in patients at high risk of cardiovascular disease. Angiology.

[21] Tseng SY, Chao TH, Li YH, Liu PY, Lee CH, Cho CL, Wu HL, Chen JH (2016). Cilostazol improves high glucose-induced impaired angiogenesis in human endothelial progenitor cells and vascular endothelial cells as well as enhances vasculoangiogenesis in hyperglycemic mice mediated by the adenosine monophosphate-activated protein kinase pathway. J Vasc Surg.

[22] Tseng SY, Chao TH, Li YH, Cho CL (2016). Cilostazol improves proangiogenesis functions in human early endothelial progenitor cells through the stromal cell-derived factor system and hybrid therapy provides a synergistic effect *in vivo*. Biomed Res Int.

[23] Biscetti F, Pecorini G, Straface G, Arena V, Stigliano E, Rutella S, Locatelli F, Angelini F, Ghirlanda G, Flex A (2013). Cilostazol promotes angiogenesis after peripheral ischemia through a VEGF-dependent mechanism. Int J Cardiol.

[24] Kawabe-Yako R, Ii M, Masuo O, Asahara T, Itakura T (2011). Cilostazol activates function of bone marrow-derived endothelial progenitor cell for re-endothelialization in a carotid balloon injury model. PLoS One.

[25] O'Donnell ME, Badger SA, Sharif MA, Young IS, Lee B, Soong CV (2009). The vascular and biochemical effects of cilostazol in patients with peripheral arterial disease. J Vasc Surg.

[26] Khera AV, Qamar A, Reilly MP, Dunbar RL, Rader DJ (2015). Effects of niacin, statin, and fenofibrate on circulating proprotein convertase subtilisin/kexin type 9 levels in patients with dyslipidemia. Am J Cardiol.

[27] Duan Y, Chen Y, Hu W, Li X, Yang X, Zhou X, Yin Z, Kong D, Yao Z, Hajjar DP, Liu L, Liu Q, Han J (2012). Peroxisome proliferator-activated receptor γ activation by ligands and dephosphorylation induces proprotein convertase subtilisin kexin type 9 and low density lipoprotein receptor expression. J Biol Chem.

[28] Sun L, Yang X, Li Q, Zeng P, Liu Y, Liu L, Chen Y, Yu M, Ma C, Li X, Li Y, Zhang R, Zhu Y (2017). Activation of adiponectin receptor regulates proprotein convertase subtilisin/kexin type 9 expression and inhibits lesions in ApoE-deficient mice. Arterioscler Thromb Vasc Biol.

[29] Sanada F, Kanbara Y, Taniyama Y, Otsu R, Carracedo M, Ikeda-Iwabu Y, Muratsu J, Sugimoto K, Yamamoto K, Rakugi H, Morishita R (2016). Induction of angiogenesis by a type III phosphodiesterase inhibitor, cilostazol, through activation of peroxisome proliferator-activated receptor-γ and cAMP pathways in vascular cells. Arterioscler Thromb Vasc Biol.

[30] Rosenzweig A (2005). Circulating endothelial progenitors-cells as biomarkers. N Engl J Med.

[31] Sponder M, Campean IA, Dalos D, Emich M, Fritzer-Szekeres M, Litschauer B, Bergler-Klein J, Graf S, Strametz-Juranek J (2017). Effect of long-term physical activity on PCSK9, high- and low-density lipoprotein cholesterol, and lipoprotein(a) levels: a prospective observational trial. Pol Arch Intern Med.

[32] Hasan AU, Ohmori K, Konishi K, Igarashi J, Hashimoto T, Kamitori K, Yamaguchi F, Tsukamoto I, Uyama T, Ishihara Y, Noma T, Tokuda M, Kohno M (2015). Eicosapentaenoic acid upregulates VEGF-A through both GPR120 and PPARγ mediated pathways in 3T3-L1 adipocytes. Mol Cell Endocrinol.

[33] Rafii S, Lyden D (2003). Therapeutic stem and progenitor cell transplantation for organ vascularization and regeneration. Nat Med.

[34] Leander K, Mälarstig A, Van't Hooft FM, Hyde C, Hellénius ML, Troutt JS, Konrad RJ, Öhrvik J, Hamsten A, de Faire U (2016). Circulating proprotein convertase subtilisin/kexin type 9 (PCSK9) predicts future risk of cardiovascular events independently of established risk factors. Circulation.

[35] Zhu YM, Anderson TJ, Sikdar K, Fung M, McQueen MJ, Lonn EM, Verma S (2015). Association of proprotein convertase subtilisin/kexin type 9 (PCSK9) with cardiovascular risk in primary prevention. Arterioscler Thromb Vasc Biol.

[36] Ridker PM, Rifai N, Bradwin G, Rose L (2016). Plasma proprotein convertase subtilisin/kexin type 9 levels and the risk of first cardiovascular events. Eur Heart J.

[37] Chen IC, Lee WH, Chao TH, Li YH, Tsai WC, Pan HA, Tseng SY, Chen JY (2012). Effects of rosiglitazone on the cardiovascular profile in postmenopausal women without diabetes mellitus: interplay of thiazolidinediones and hormone therapy. Menopause.

[38] Chen IC, Chao TH, Tsai WC, Li YH (2010). Rosiglitazone reduces plasma levels of inflammatory and hemostatic biomarkers and improves global endothelial function in habitual heavy smokers without diabetes mellitus or metabolic syndrome. J Formos Med Assoc.

